# Paradoxical immune reconstitution inflammatory syndrome due to toxoplasmic encephalitis: two cases and review of initiation of antiretroviral timing in toxoplasmic encephalitis IRIS

**DOI:** 10.12688/f1000research.2-133.v1

**Published:** 2013-05-30

**Authors:** Andrew R DiNardo, Douglas Smoot Lewis, Hoonmo L Koo, J Clay Goodman, Elizabeth Chiao, Roberto Andrade

**Affiliations:** 1 Department of Medicine, Baylor College of Medicine, Houston, TX, 77030, USA; 2 Department of Pathology, Baylor College of Medicine, Houston, TX, 77030, USA

## Abstract

*Toxoplasma* encephalitis immune reconstitution inflammatory syndrome ****(TE-IRIS) is rare and usually occurs in an unmasking, rather than paradoxical form. To the best of our knowledge, only two cases of paradoxical TE-IRIS and nine cases of unmasking TE-IRIS have been previously described. We present two additional cases of histopathology-consistent paradoxical TE-IRIS, after early initiation of antiretroviral therapy (ART), and review the literature on TE-IRIS. Three of the four reported cases of paradoxical TE-IRIS were associated with early (within one week) initiation of ART, an issue that was not addressed in the 2009 US Department of Health and Human Services guidelines for the treatment of opportunistic infections.

## Case 1

**Figure 1.**
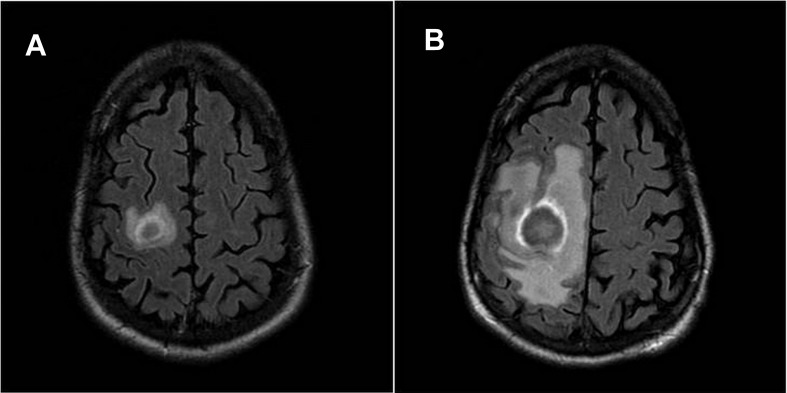
Brain MRI of patient 1 at presentation showed an ill-defined 1 cm right medial occipitotemporal gyrus mass and a 1.8×1.7×1.5 cm mass in the subcortical white matter of the right precentral gyrus with surrounding edema ( **A**). After 14 days of anti-toxoplasma treatment and antiretroviral therapy, a follow-up MRI revealed enlargement of the prior right precentral gyrus lesion now to maximum size of 2.2 cm ( **B**).

A 35 year-old man with AIDS, not on antiretroviral therapy (ART) or prophylaxis, presented with shortness of breath, productive cough and fever for one week, as well as progressive weakness of his left upper extremity over the previous two weeks. His exam was significant for weakness (4/5) in his left-hand grip. His CD4 count was 8 (1%) cells/µl and his viral load was 547,000 copies/ml. Serum *Toxoplasma* IgG levels were undetectable. His chest X-ray revealed a right middle lobe infiltrate and three sputum samples for acid fast bacilli (AFB) were negative. Respiratory symptoms resolved with ceftriaxone and azithromycin. An MRI of his brain revealed two ring-enhancing lesions in the right precentral and occipital temporal areas ( Figure 1A). The patient refused a lumbar puncture and was empirically started on anti-toxoplasmic therapy (pyrimethamine, sulfadiazine and leucovorin) on day two. On day four, the patient was started on ART with abacavir, lamivudine and raltegravir.

**Figure 2.**
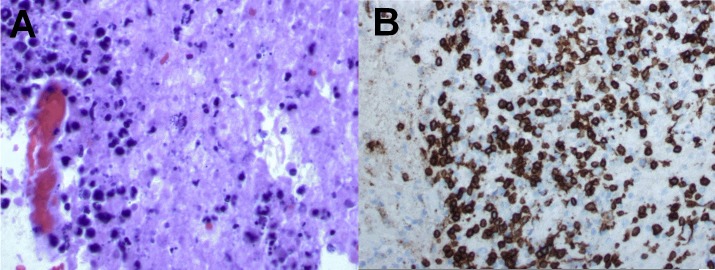
Brain histopathology of patient 1. Significant necrosis with perivascular mixed inflammatory cells, with lymphocyte predominance and concentric vascular wall fibroblastic proliferation. Rare individual *Toxoplasma gondii* bradyzoites and numerous tachyzoites were identified (100X) ( **A**). Immunophenotyping of the inflammatory cells, showing a mixture of mature T- and B-lymphocytes with CD8+ over CD4+ cell predominance. There was no evidence of other infections or CNS lymphoma (100X) ( **B**).

Over the next two weeks, the patient’s function deteriorated: his left deltoid muscle strength weakened to 2/5 (previously 5/5), he developed a left facial droop and left hip flexor and quadriceps weakness were 3/5 (previously 5/5). He consented to a lumbar puncture and his CSF revealed 6 white blood cells (WBC) per mm3 (96% lymphocytes and 4% monocytes), glucose of 41 g/L and protein of 92 g/L. EBV PCR in the CSF was positive. On hospital day ten, he suffered a tonic-clonic seizure. A repeat MRI on day 14 of empiric anti- *Toxoplasma* therapy, showed enlargement of the two prior lesions and development of a third lesion ( Figure 1B). After twenty days of anti-toxoplasmic therapy, a brain biopsy revealed rare *Toxoplasma gondii* tachyzoites and numerous bradyzoites ( Figure 2A), as well as CD8+ predominant lymphocytic infiltrates ( Figure 2B). A repeat CD4 count and viral load measure was 13 cells/µll (1%) and 10,300 copies/ml respectively. Anti- *Toxoplasma* treatment and ART were continued and the patient was started on corticosteroids (dexamethasone 4 mg every 4 hours tapered to prednisone 10 mg per day over 2 weeks), with gradual improvement but not complete resolution of his symptoms and signs.

## Case 2

A 51 year-old man presented with two weeks of fevers, weight loss, frontal headaches, unsteady gait and upper extremity left-sided weakness. Three weeks prior, he had been diagnosed with AIDS and was not on ART or prophylaxis. On exam, he had left deltoid and hand-grip weakness (3/5) and left hip flexor and quadriceps extremity weakness (4/5), as well as decreased light sensation in his left arm.

**Figure 3.**
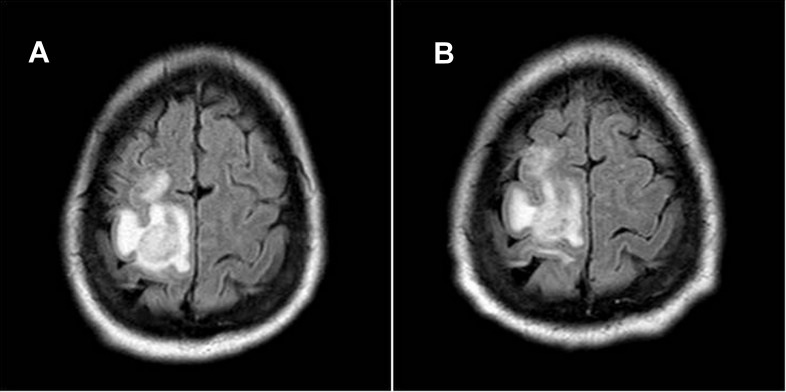
Brain MRI of patient 2 at presentation showed an irregular ring-enhancing lesion in the right frontoparietal region with maximal transverse dimension of 2.2 cm ( **A**). After 10 days of anti-toxoplasma treatment and antiretroviral therapy, follow-up MRI revealed no significant interval change of enhancing frontoparietal lesion ( **B**).

His CD4 count was 152 (13%) cells/µl and his viral load was 433,000 copies/ml. *Toxoplasma* IgG was positive. An MRI revealed multiple ring-enhancing lesions in his frontoparietal region ( Figure 3A). CSF showed 6 WBCs (100% lymphocytes), undetectable JC virus DNA and no malignant cells on cytopathology or flow cytometry. On day two, the patient was started on pyrimethamine, sulfadiazine and leucovorin, as well as lopinavir-ritonavir, tenofovir and emtricitabine.

**Figure 4.**
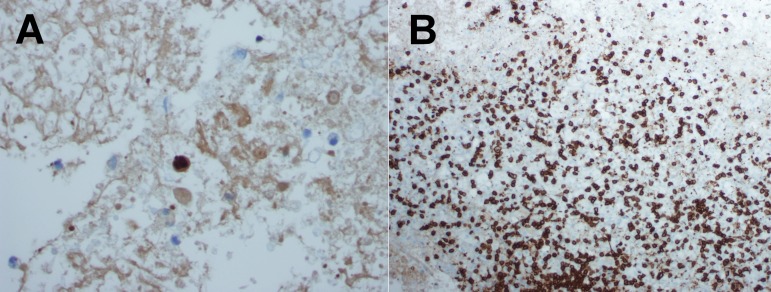
Patient 2 H&E staining necrotic tissue with scattered inflammatory cells consisting of mature lymphocytes and macrophages and some perivascular lymphocytes were present (not shown). Immunohistochemistry for *T. gondii* (400X) ( **A**). Immunophenotyping of the inflammatory cells, showing again CD8+ over CD4+ T-cell predominance (100X) ( **B**).

The patient experienced improvement in his weakness and gait to near-baseline and was discharged to rehabilitation. Ten days after initiation of treatment, the patient’s headache and left-sided weakness returned, in addition to a new left-sided facial droop. A repeat MRI showed no significant change ( Figure 3B). A repeat CD4 count was 133 (15%) cells/µl and his viral load was 77,700 copies/ml. A brain biopsy revealed *T. gondii* by immunohistochemistry ( Figure 4A) and a CD8+ lymphocytic infiltrate ( Figure 4B). His symptoms progressively improved with continued anti- *Toxoplasma* therapy, ART and a corticosteroid taper (initially dexamethasone 4 mg every 6 hours for days, tapered to oral prednisone 2.5 mg per day, over a six-week period).

## Discussion

Despite *Toxoplasma* encephalitis being the most common cause of focal brain lesions in AIDS patients in resource-rich countries ^1^, TE-IRIS is rare ^2, 3^ due to the elegant mechanisms of immune evasion by *T. gondii* ^4^. A delicately controlled balance between host immune response and parasite immune evasion characterizes the life cycle of *T. gondii*. Bradyzoites survive in a dormant intracellular state in a parasitophorous vacuole that, typically, evades intracellular acidification. The tissue cysts are kept in check by a Th1 predominant immune response ^5^ characterized by macrophages and dendritic cells, producing IL-12, triggering CD4 cells to release IL-2, IFN-γ, IL-6 and TNF-α, thereby stimulating a reactive oxygen and nitric oxide-mediated cytotoxic response.

AIDS patients have impaired IFN-γ and IL-2 production that leads to uncontrolled *T.* *gondii* growth and abscess formation with necrotic centers and hyperemic edges, which appear as the characteristic multiple ring-enhancing lesions on MRI ( Figure 1 and Figure 3) ^6^. Initiation of ART and subsequent immune reconstitution restores the ability to control *Toxoplasma* ^7^. Pathologic excessive reactivation of the immune system is evident on histopathology by an exuberant lymphocytic infiltration with CD8+ predominance, which is not otherwise seen in TE and is characteristic of TE-IRIS ( Figure 2 and Figure 4) ^4^.

For most opportunistic infections, there is increasing evidence to support early initiation of ART ^8^. However, in the case of severe OIs involving the central nervous system (CNS) there is also evidence supporting delayed initiation of ART. In a retrospective review of 514 patients with HIV and tuberculosis meningitis, there was significant mortality in those started on ART and anti-tuberculosis therapy simultaneously ^9^. In a randomized, double-blinded, placebo-controlled prospective study of tuberculosis meningitis there was significant morbidity associated with immediate initiation of ART ^10^. Recently, the prospective COAT (cryptococcal optimal ART timing) trial, designed to look at the optimal timing of ART initiation in cryptococcal meningitis, was stopped early due to increased mortality with early initiation of ART ^11^. Since TE-IRIS is very rare, there is a paucity of data regarding the timing of ART initiation; therefore this issue is not addressed in the current US Department of Health and Human Services guidelines ^12^.

Immune reconstitution inflammatory response syndrome cases are classified as either unmasking (unmasking of an occult infection not known at the time of ART initiation) or paradoxical (the paradoxical worsening of a known disease despite appropriate therapy). In their case series of TE-IRIS, Martin-Blondel *et al.* report three cases of unmasking TE-IRIS and 65 cases of TE that did not experience paradoxical TE-IRIS despite virologic and immunologic response ^2^. All three patients had unmasking TE-IRIS (none of them had been diagnosed with TE before the initiation of ART) and they all developed IRIS more than a month after the initiation of ART. None of the 65 patients with TE who did not develop IRIS had received ART before two weeks of anti-toxoplasma treatment (Martin-Blondel personal communication). To our knowledge, we describe only the third and fourth cases of paradoxical TE-IRIS in the literature ^3, 13^. In both of our patients, as well as the case of paradoxical TE-IRIS presented by Tremont-Lukats ^3^, ART was started simultaneously or within the first week of anti-toxoplasmic therapy. In the case of paradoxical TE-IRIS described by Cabral *et al.* ^13^, ART was delayed for twenty days after initiation of anti-toxoplasmic therapy (Ferracini personal communication).

In conclusion, we present two patients with AIDS and CNS toxoplasmosis, who had disease progression, despite empiric anti-toxoplasmic therapy and a 1-log decrease in their HIV viral load with ART. Since the overwhelming majority of patients with toxoplasmic encephalitis improve after two weeks of anti-toxoplasma therapy ^14^, the worsening symptoms led to a brain-biopsy. In both patients, histopathology showed a vigorous cytotoxic immune response, consistent with TE-IRIS ^1, 4^. Both patients improved with continuation of anti-parasitic treatment, ART and high-dose steroids. In these two cases and one of the two previous cases of paradoxical TE-IRIS, initiation of ART took place simultaneous or within the first week of anti-parasitic therapy. While generalizations cannot be made with only four cases, until further data evaluates the issue, we recommend very close monitoring if ART is started before at least two weeks of anti-toxoplasma therapy. Moreover, our cases illustrate that, for CNS toxoplasmosis in AIDS patients who have been recently started on ART, after performing a biopsy, the threshold to administer steroids for possible IRIS should be low, if there is any evidence of clinical deterioration.

## Consent

Informed consent for publication of the clinical details and clinical images was obtained from the patients.
