# Supplementary material for: Paradoxical immune reconstitution inflammatory syndrome due to toxoplasmic encephalitis: two cases and review of initiation of antiretroviral timing in toxoplasmic encephalitis IRIS
Source: F1000Res. 2013 May 30;2:133. [Version 1] doi: 10.12688/f1000research.2-133.v1 (PMC3814919; doi:10.12688/f1000research.2-133.v1)
Supplement: Peer review history

# Peer review: Referee response for version 1

**Published:** 2013-07-30

**Authors:** Guillaume Martin-Blondel

**Affiliations:** 1 Infectious and Tropical Diseases Department, Toulouse University Hospital, University Toulouse III, Toulouse, France

In general, earlier HAART improves outcome particularly in those with the lowest CD4 counts. However, opportunistic infections of the CNS such as TB and cryptococcosis are an exception, and timing of HAART requires special consideration. The optimal timing of HAART in the setting of toxoplasma encephalitis has not been studied. This well written and informative case report illustrates that initiating HAART (very) early after beginning anti-toxoplasma therapy might be deleterious, deserving further studies.

I have read this submission. I believe that I have an appropriate level of expertise to confirm that it is of an acceptable scientific standard.

# Peer review: Referee response for version 1

**Published:** 2013-05-31

**Authors:** Matthijs Brouwer

**Affiliations:** 1 Department of Neurology, Academic Medical Center, University of Amsterdam, Amsterdam, Netherlands

This is a well written and interesting observation that is helpful for doctors treating HIV-infected patients. Although the number of patients is low with TE-IRIS, one could argue to delay HAART until the patient has been treated for 2 weeks with anti-toxoplasmosis treatment, which is an important message.

I have read this submission. I believe that I have an appropriate level of expertise to confirm that it is of an acceptable scientific standard.

# Peer review: Referee response for version 1

**Published:** 2013-05-31

**Authors:** Bruce Brew

**Affiliations:** 1 Department of Neurology, St Vincent's Hospital, Sydney, NSW, Australia

This is a useful addition to the literature and serves as a cautionary note to clinicians. There are some minor issues: the grading scale for weakness is the MRC scale and the viral load referred to in the first patient relates to HIV.

I have read this submission. I believe that I have an appropriate level of expertise to confirm that it is of an acceptable scientific standard.

# Response

**Published:** 2013-06-05

**Authors:** Andrew DiNardo

**Affiliations:** Baylor College of Medicine, USA

Thank you for your comments. Yes, the strength scale is MRC & yes the "viral load" was meant to imply "HIV viral load."
